# Disease-Specific Autoantibodies Induce Trained Immunity in RA Synovial Tissues and Its Gene Signature Correlates with the Response to Clinical Therapy

**DOI:** 10.1155/2020/2109325

**Published:** 2020-10-06

**Authors:** Xiaoli Dai, Xiaoqiu Dai, Zheng Gong, Chen Yang, Keqin Zeng, Fang-Yuan Gong, Qiao Zhong, Xiao-Ming Gao

**Affiliations:** ^1^Department of Clinical Laboratory, The Second Affiliated Hospital of Soochow University, Suzhou 215009, China; ^2^Institute of Biology and Medical Sciences, Soochow University, Suzhou 215123, China; ^3^Department of Laboratory Medicine, The Affiliated Suzhou Hospital of Nanjing Medical University, Suzhou 215002, China; ^4^Department of Rheumatology, The First Affiliated Hospital of Soochow University, Suzhou 215002, China

## Abstract

Much evidence suggests that trained immunity is inappropriately activated in the synovial tissue in rheumatoid arthritis (RA), but the underlying mechanism remains unclear. Here, we describe how RA-specific autoantibody deposits can train human monocytes to exert the hyperactive inflammatory response, particularly via the exacerbated release of tumor necrosis factor *α* (TNF*α*). Comparative transcriptomic analysis by plate-bound human IgG (cIgG) or *β*-glucan indicated that metabolic shift towards glycolysis is a crucial mechanism for trained immunity. Moreover, the cIgG-trained gene signatures were enriched in synovial tissues from patients with ACPA- (anticitrullinated protein antibody-) positive arthralgia and undifferentiated arthritis, and early RA and established RA bore a great resemblance to the myeloid pathotype, suggesting a historical priming event *in vivo*. Additionally, the expression of the cIgG-trained signatures is higher in the female, older, and ACPA-positive populations, with a predictive role in the clinical response to infliximab. We conclude that RA-specific autoantibodies can train monocytes in the inflamed lesion as early as the asymptomatic stage, which may not merely improve understanding of disease progression but may also suggest therapeutic and/or preventive strategies for autoimmune diseases.

## 1. Introduction

The dogma that only adaptive immunity can build immunological memory has recently been challenged by the new concepts of trained immunity or innate immune memory [1, 2]. This notion was first described for exposure to *Candida albicans* or *β*-glucans, which can protect the host against reinfection via functional reprogramming of monocytes. Mechanistically, this process has been defined as sustained remodeling in transcriptional programs that does not involve permanent genetic changes, such as gene mutation, alternative splicing, or recombination [2]. Moreover, there is a metabolic shift towards glycolysis during the induction of trained immunity [3]. An *in vitro* experimental trained model showed that this can lead to increased responsiveness upon secondary stimulation by pathogens [4], demonstrated by the enhanced production of inflammatory mediators and the capacity for eliminating the infection. However, inappropriate activation of trained immunity may cause a series of deleterious consequences via its hyperactivity. Intriguingly, a growing body of evidence suggests that this hyperactivity may occur as an early event during the asymptomatic stage of disease, such as hyperuricemia and hyperlipoproteinemia [5, 6].

*Rheumatoid arthritis* (RA) is one of the most common autoimmune disease and is characterized by synovial inflammation leading to cartilage damage and bone destruction [7]. Early recognition of the characteristics of inflammatory arthritis may provide a therapeutic window of opportunity for avoiding irreversible joint damage. Importantly, increased synovial macrophages in the sublining are a hallmark of early RA [8, 9], which may drive T lymphocyte infiltration via antigen presentation and further trigger B lymphocyte expansion and the production of immunoglobulin (Ig) and other inflammatory mediators. Through a combined histopathological and transcriptomic approach, synovial tissue can be classified into three major pathotypes with specific gene signatures in RA, namely, the fibroid, myeloid, and lymphoid pathotypes, which correspond to their cellular composition [10, 11]. By using gene set enrichment analysis (GSEA) to identify the characteristics of infiltrating immune cells, these signatures can improve the clinical classification and therapeutic response in patients with early-stage inflammatory arthritis [10]. Notably, several clues suggest that trained immunity may occur under the inflamed synovial lesion, leading to the asymptomatic transition to inflammatory arthritis [5, 6, 12]. The fractionated synovial macrophages from patients with highly active RA also exhibit hyperactive inflammatory responsiveness, which produces larger amounts of inflammatory cytokines, represented by tumor necrosis factor *α* (TNF*α*) [9]. Therapy targeting these macrophage-produced cytokines has proven to be successful. Furthermore, etanercept and adalimumab can downregulate histone tail trimethylation and histone 3 and 4 acetylation at the promoter region of *CCL2* (MCP-1) in monocytes, which correlates with the disease activity in RA [13]. Moreover, glycolysis in RA synovial macrophages is upregulated, as are the rate-limiting enzymes (e.g., pyruvate kinase (PKM) and hexokinase (HK)), which present as signs of increased metabolic activity [14, 15]. However, the underlying mechanism of trained immunity in RA remains unclear.

Recently, we reported that the deposits of IgG immune complexes (ICs) can sensitize human monocytes to a hyperactive inflammatory response accompanied by transcriptomic and epigenetic changes, which is closely reflected in clinical RA [16]. In the synovium, abundant anticitrullinated protein antibodies (ACPA) and rheumatoid factor (RF) formed ICs with antigens, resulting in the activation of their receptors for the Fc fragment of IgH chains (FcRs) [17]. However, the resulting TNF*α* levels were rather low, indicating the marginal capacity of Fc*γ*R (Fc gamma R) for inducing cytokine production when stimulated without any costimulation. The arthritogenic autoantibodies, such as anti-lactoferrin, correlate well with disease activity in RA, also displaying an extraordinary inflammatory response *in vitro* [18]. The inflammatory property of arthritogenic autoantibodies can be explained by the “two-hit” hypothesis, which induces secondary Toll-like receptor (TLR) signaling to Fc*γ*R from their antigens (e.g., vimentin, fibrinogen, and lactoferrin) [18–20]. Moreover, ACPA/RF double-positive patients with arthralgia may quickly develop RA, which suggests that the serological response is a high risk for disease progression [21]. Therefore, we hypothesize that disease-specific autoantibodies may induce trained immunity, which may be reflected in RA synovial tissue.

In the present study, RA-specific autoantibodies that could induce trained immunity were defined using an *in vitro* experimental model. The gene expression profile of plate-bound IgG- (cIgG-) trained monocytes was generated and compared with the paradigm of trained immunity induced by *β*-glucan and showed significant enrichment for glycolysis, suggesting that metabolic shift is a key mechanism in trained immunity. Furthermore, we performed GSEA to assess the cIgG-trained gene signatures against the transcriptome of synovial tissues from patients with ACPA-positive arthralgia, undifferentiated arthritis, early RA, established RA, and osteoarthritis and from healthy donors. Surprisingly, these cIgG-trained signatures resembled those of the myeloid pathotype and were associated with enhanced immune cell infiltration in the synovial tissue, which indicated a historical priming event *in vivo*. Additionally, the expression of cIgG-trained signatures has bias on sex, age, and ACPA status and played a predictive role in the clinical response to infliximab. We conclude that RA-specific autoantibodies can train monocytes in the inflamed lesion as early as the asymptomatic autoimmunity stage, which may not merely improve understanding of disease progression but may also suggest therapeutic and/or preventive strategies for autoimmune diseases.

## 2. Materials and Methods

### 2.1. Purification of ACPA IgG and RF IgM in Human Serum

ACPA IgG and RF IgM were purified by sequential affinity chromatography as previously described [22]. Briefly, ACPA and RF double-positive serum samples were pooled from 30 RA patients with high titers of ACPA and RF who presented at the Department of Rheumatology of the First Affiliated Hospital of Soochow University, Suzhou, China. Before affinity purification, IgG was purified from the serum by a HiTrap Protein G column (GE Healthcare). After washing with 12 column volumes of phosphate buffer containing 1 M NaCl, preelution was performed to dissociate RF-IgG interaction before the high-affinity interaction between protein G and IgG was disrupted by elution with 0.2 M glycine-HCl (pH 2.7). CCP (cyclic citrullinated peptide) was synthesized and conjugated to cyanogen bromide- (CNBr-) activated Sepharose™ 4B (GE Healthcare) according to the manufacturer's instructions. The purified IgG was then applied to the CCP columns and refined at a rate of 1 ml/min. The bound antibodies (Abs) were washed extensively with phosphate-buffered saline (PBS), eluted using 0.2 M glycine-HCl (pH 2.7), and neutralized with 1 M Tris-HCl (pH 8.0). ACPA IgG was concentrated with ultrafiltration using Amicon Ultra Centrifugal Filter Units (Merck Millipore). The IgG-depleted RA serum pools were used to purify the IgM using the CaptureSelect™ IgM Affinity Matrix (Thermo Scientific). RF IgM was obtained by loading on human IgG Sepharose™ 6B (GE Healthcare). The RF IgM fractions were collected and neutralized by adding 1 M Tris-HCl (pH 8.0) immediately after collection. The RF IgM was then concentrated using ultrafiltration. Both the ACPA IgG and RF IgM fractions were depleted of endotoxin by filtration through a polymyxin B column (Thermo Scientific). Lipopolysaccharide (LPS) levels were confirmed to be <0.058 ng/ml using a highly sensitive LPS enzyme-linked immunoassay (ELISA) kit (Cloud-Clone).

### 2.2. Monocyte Isolation by Magnetic Separation

Peripheral blood mononuclear cells (PBMCs) from healthy donors were isolated using centrifugation with Ficoll-Paque (Sigma-Aldrich). Human monocytes were purified from the PBMCs using anti-human CD14-labeled magnetic beads (Miltenyi Biotec) according to the manufacturer's instructions. Flow cytometry and microscopic observation showed that >95% of the purified monocytes were CD14-positive and viable. The monocytes were cultured in RPMI 1640 medium supplemented with 10% (*v*/*v*) autologous serum from corresponding donors and with penicillin/streptomycin (100 U/ml), at 37°C in 5% CO_2_.

### 2.3. *In Vitro* Trained Immunity Model with RA Autoantibodies

**Figure 1:**
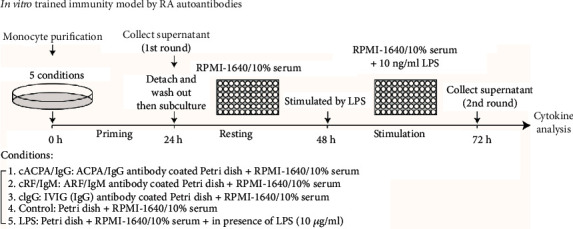
Schematic overview of *in vitro* trained immunity model by RA autoantibodies. Freshly isolated CD14+ human monocytes were cultured for 24 h in RPMI 1640 medium in Petri dishes precoated with 10 *μ*g/ml purified ACPA IgG (cACPA/IgG), RF IgM (cRF/IgM), or IVIG (cIgG). Control cells were cultured with RPMI 1640 medium or in the presence of LPS (1 *μ*g/ml). After priming, the monocytes were detached, washed, and subcultured in 96-well plates for resting. Then, the Mo(cACPA/IgG), Mo(cRF/IgM), Mo(cIgG), Mo(control), and Mo(LPS) were stimulated by LPS (10 ng/ml) for 24 h. Cytokines and chemokines in the supernatants from either the priming or stimulation phase were measured by ELISA.

Freshly purified human monocytes from six unrelated healthy donors were incubated with RPMI 1640 culture medium in 10 cm Petri dishes precoated with 10 *μ*g/ml ACPA IgG, RF IgM, or intravenous Ig (IVIG) (IgG) (SinoPharm) for 24 h. Control cells were cultured with medium alone or in the presence of LPS (1 *μ*g/ml) (Escherichia coli 0111:B4, Sigma-Aldrich) (Figure 1). Then, the cells were detached with 0.25% trypsin containing 0.9 mM EDTA (Invitrogen), washed with warm PBS, and then subcultured in 96-well plates at 2 × 10^5^ cells/well. After resting, the cells were restimulated with or without RPMI 1640 medium and LPS (10 ng/ml) for 24 h. The first-round (priming) and second-round (stimulation) supernatants were collected and stored at -20°C until measurement.

For intracellular staining, 5 × 10^6^ monocytes were primed in 10 cm Petri dishes with or without IgG coating (10 *μ*g/ml) for 24 h and then restimulated with LPS (10 ng/ml) for 2 h. Staining was performed using the Intracellular Staining Kit (eBioscience) with allophycocyanin- (APC-) conjugated mouse anti-human TNF*α* (BioLegend) or mouse IgG1 as the isotype Ab. After washout, the fluorescence was assessed by flow cytometry, and the mean fluorescence intensity (MFI) was calculated.

### 2.4. Enzyme-Linked Immunosorbent Assays

The cytokine and chemokine concentrations were determined using commercial ELISA kits for human TNF*α*, interleukin-6 (IL6), CXCL8 (all, Invitrogen), and CCL2 (R&D Systems). Standard curves were established using recombinant human cytokines/chemokines; the assay detection sensitivity was 4 pg/ml for TNF*α*, 7.8 pg/ml for IL6, 2 pg/ml for CXCL8, and 10 pg/ml for CCL2.

### 2.5. RNA Sequencing

RNA sequencing was performed as previously described [16]. Briefly, cellular RNA was extracted from the cells, poly(A)-enriched, fragmented, and converted into an Illumina-compatible sequencing template library. Single-end sequencing on an Illumina HiSeq 2000 system was carried out by the Beijing Genomics Institute (BGI, Wuhan, China). Gene expression was quantified using RNA Sequencing by Expectation Maximization (RSEM) [23]. Differentially expressed genes (DEGs) were compared between groups via Empirical Bayes (EB) methods for RNA sequencing (EBSeq) [24]. Kyoto Encyclopedia of Genes and Genomes (KEGG) enrichment analysis of the DEGs was performed using clusterProfiler [25]. The RNA sequencing data reported here have been deposited into Gene Expression Omnibus (https://www.ncbi.nlm.nih.gov/geo/) under accession number GSE102728.

### 2.6. Flow Cytometry

For cellular phenotype analysis, the monocytes were collected and washed with PBS, and the pellets were incubated for 30 min at 4°C with phycoerythrin- (PE-) conjugated mouse anti-human CD14, CD16, CD209, CD40, and CD80 or APC-conjugated mouse anti-human CD32, CD1b, and CD86 or fluorescein isothiocyanate- (FITC-) conjugated mouse anti-human CD64 or PE-, APC-, and FITC-conjugated isotype control Abs (BioLegend). After washing, the cells were analyzed using a BD FACSCanto™ II flow cytometer (BD Biosciences).

### 2.7. Transcriptomic Cohort of Synovial Tissues

The transcriptomes of a synovial biopsy cohort were retrieved from Gene Expression Omnibus (accession number GSE89408). The raw RNA sequencing data were processed and quantified by GEO RNA-seq Experiments Interactive Navigator (GREIN) [26]; the clinical information was obtained from a previous study [27] and is listed in Supplementary Table 1. Briefly, healthy donors and osteoarthritis patients were identified from a group of patients with knee pain attending a sports medicine day at a surgical facility, which was distinguished by the evidence of arthritis or clinical history. Arthralgia patients refer to a population based on their symptoms, rather than a specific diagnosis, while undifferentiated arthritis was defined as clinical signs of synovitis and patients that did not meet the 2010 American College of Rheumatology criteria for RA. Early RA refers to treatment-naïve RA within 12 months of first assessment; established RA refers to treatment-experienced RA of >12-month disease duration. The Institution Ethics Review board approved all protocols for collecting synovial biopsies. All patients signed the consent form for participating in the study.

### 2.8. Gene Set Enrichment Analysis

Hallmark gene sets (*n* = 50) in the Molecular Signatures Database (MSigDB) [28] were used for comparative transcriptomic analysis with *β*-glucan-trained monocytes (accession number GSE58310) [2], which was performed using GSEA [29]. The cIgG gene signatures were defined from the top 100 upregulated genes among the DEGs by RNA sequencing of cIgG-trained monocytes. In addition, the gene sets of immune tissue cells, including CD4+ T cells, CD8+ T cells, dendritic cells, CD19+ B cells, T regulatory (Treg) cells, basophils, eosinophils, neutrophils, CD34+ progenitors, synoviocytes, and fibroblasts, were described as done previously [30], which was based on cap analysis gene expression (CAGE) data from the Functional Annotation of the Mammalian Genome (FANTOM5) project [31]. Additionally, the M1 macrophage gene signatures were defined based on the gene expression of in vitro-stimulated monocytes that underwent classical activation (M1) with LPS and interferon gamma (IFN*γ*) versus alternative activation (M2) with IL4 [32]. Pathotype-specific gene sets for the myeloid, lymphoid, and fibroid phenotypes were defined by the study on the Michigan RA cohort [11]. Supplementary Table 2 lists the information on the gene sets.

### 2.9. Statistical Analysis

All statistical analyses were performed with Prism software (GraphPad). As the normal distribution of the samples could not be assumed, the Kruskal–Wallis test followed by post hoc testing by Dunn's multiple comparison test was used for >2 groups, and the two-tailed Mann–Whitney *U* test was used for two groups of unpaired samples. A *P* value < 0.05 was considered statistically significant.

### 2.10. Study Approval

The Soochow University Ethics Committee approved this study. The methods were carried out in accordance with the guidelines of Soochow University. Written informed consent was obtained from all participants prior to inclusion in the study.

## 3. Results

### 3.1. ACPA IgG Deposits Induced Trained Immunity in Human Monocytes

**Figure 2:**
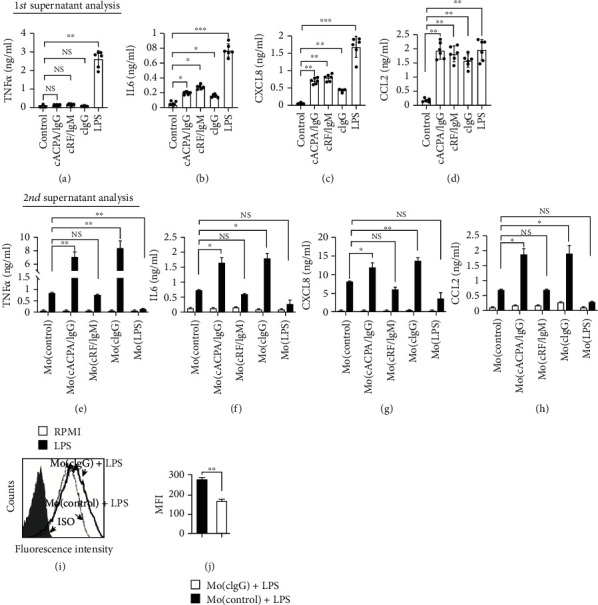
Enhanced LPS response by ACPA IgG-primed monocytes. (a–d) ELISA quantification of TNF*α*, IL6, CXCL8, and CCL2 in the supernatant during monocyte priming. After priming, the Mo(cACPA/IgG), Mo(cRF/IgM), Mo(cIgG), Mo(control), and Mo(LPS) were treated with LPS (10 ng/ml); monocytes treated in RPMI 1640 medium were used as the control (RPMI). (e–h) ELISA quantification of TNF*α*, IL6, CXCL8, and CCL2. Each dot represents one donor (*n* = 6). (i) The Mo(cIgG) and Mo(control) were stimulated with LPS (10 ng/ml) for 2 h, followed by intracellular staining of TNF*α*. (j) Cells stained with isotype control Abs (filled histogram) were used as negative controls. The MFI of TNF*α* was compared. Data (mean ± SEM) are from three independent experiments. NS: no statistical significance; ∗*P* < 0.05, ∗∗*P* < 0.01, and ∗∗∗*P* < 0.001. The Kruskal–Wallis test was performed, followed by post hoc testing by Dunn's multiple comparison test, for comparing >2 groups. The Mann–Whitney *U* test was used for comparing two groups.

To test our hypothesis that the presence of RA-specific autoantibodies can train innate immunity, we purified ACPA IgG to investigate the capacity for trained immunity using an in vitro model based on a previous study [4] (Figure 1). As the presence of ACPA IgG probably forms ICs against citrulline proteins, plate-bound RA autoantibodies were used to mimic the complex crosslinking of FcR in vivo. Freshly purified monocytes from six unrelated healthy donors were incubated with cACPA IgG on a Petri dish for 24 h priming, followed by cytokine and chemokine quantitation in the first-round culture supernatant. Interestingly, the cACPA IgG exhibited limited TNF*α* and IL6 production of <200 pg/ml (Figures 2(a) and 2(b)). In contrast, LPS priming strongly stimulated the release of proinflammatory cytokines by the monocytes in the form of TNF*α* and IL6. In addition, the plate-bound RA autoantibodies stimulated the release of substantial levels of chemokines by monocytes, including CXCL8 and CCL2 (Figures 2(c) and 2(d)), which may provide chemotactic signaling for the infiltration of myeloid lineage cells into the inflamed lesion [33]. Notably, there was no significant difference in the release of inflammatory mediators during the priming of the monocytes by cACPA IgG, cRF IgM, or cIgG.

Then, the cells were detached with 0.25% trypsin-EDTA and washed with warm PBS to remove the previous exposure. With accurate cell counting, the primed cells were subcultured into 96-well plates without additional treatment. After another 24 h rest, the cells were stimulated with 10 ng/ml LPS (Figure 1). As expected, the LPS induced an endotoxin tolerance phenotype on the monocytes, demonstrated as attenuated cytokine and chemokine release (Figures 2(e)–2(h)). Strikingly, both cACPA IgG-primed monocytes (Mo(cACPA/IgG)) and cIgG-primed monocytes (Mo(cIgG)) displayed an increased inflammatory response compared with the control (Mo(control)) and cRF IgM-primed monocytes (Mo(cRF/IgM)) in terms of the amount of cytokines or chemokines released following LPS stimulation (Figures 2(e)–2(h)). This consistency between Mo(cACPA/IgG) and Mo(cIgG) suggested that the IgG isotype Abs, but not IgM, triggered trained signaling. Importantly, exposing the primed cells to RPMI 1640 medium did not induce a significant amount of inflammatory mediators, which may have excluded the accumulative effect of cytokine release. Additionally, the increase in TNF*α* (about 10-fold) was much greater than that of the other cytokines or chemokines (2- to 4-fold) between the Mo(cACPA/IgG) and Mo(control). Furthermore, we performed intracellular staining of TNF*α* synthesis on the Mo(cIgG). Notably, TNF*α* fluorescence intensity following LPS stimulation was sharply increased on the Mo(cIgG) as compared with the Mo(control) (Figures 2(i) and 2(j)). These findings suggest that the IgG autoantibody deposits in synovial tissue may induce trained immunity in vivo, which may increase inflammatory cytokine release and contribute to the pathogenesis of RA.

### 3.2. Gene Expression Profile of cIgG-Trained Monocytes

**Figure 3:**
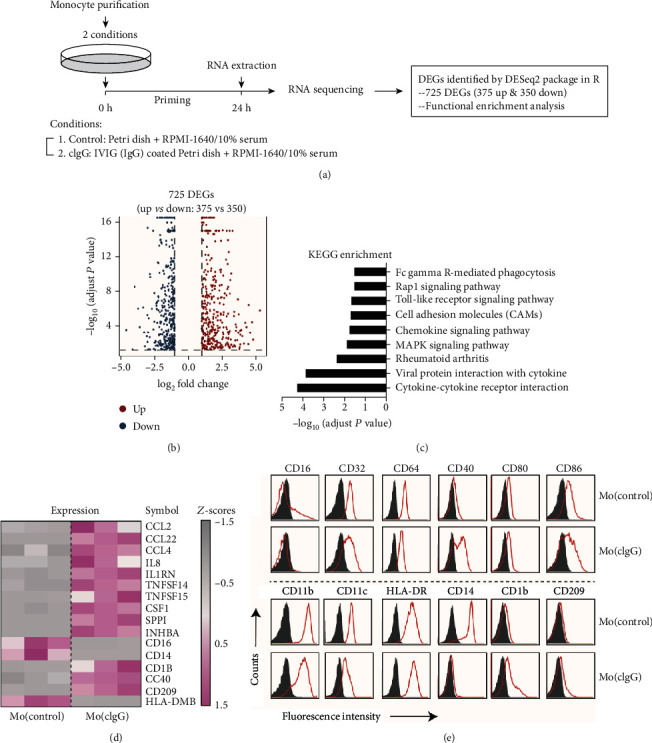
Specific gene signatures of cIgG-trained human monocytes. (a) Schematic workflow of RNA sequencing and analysis of the cIgG-trained monocytes. (b) Volcano plot of 725 DEGs from Mo(cIgG) as compared with Mo(control). (c) Bar plot of KEGG enrichment analysis of DEGs in Mo(cIgG). (d) Heatmap showing the signature cytokine, chemokine, and cellular marker genes expressed in Mo(cIgG) and Mo(control). (e) Flow cytometric analysis of Mo(cIgG) stained with isotype control Abs (filled histogram) and specific markers as indicated (red).

We conducted transcriptomic analysis of the cIgG-trained monocytes to examine the gene expression profile of immunity trained by the RA autoantibodies. Briefly, freshly fractionated monocytes from three healthy donors were treated according to the in vitro training model (Figure 3(a)). After 24 h priming, the cellular RNA was extracted and underwent high-throughput RNA sequencing for transcriptomic analysis. EBSeq package (v 3.10) [24] showed that, in total, there were 725 DEGs between the Mo(cIgG) and Mo(control): 375 were upregulated and 350 were downregulated (Figure 3(b)). KEGG enrichment analysis of the DEGs was performed to determine the characteristics of the Mo(cIgG) and showed that the DEGs were enriched for cytokine-cytokine receptor interaction, viral protein interaction with cytokine, RA, and the MAPK signaling pathway, chemokine signaling pathway, cell adhesion molecules (CAMs), Toll-like receptor signaling pathway, Rap1 signaling pathway, and Fc*γ*R-mediated phagocytosis (Figure 3(c)).

Figure 3(d) shows the strongly upregulated or downregulated genes in the Mo(cIgG). In line with the functional findings, the Mo(cIgG) displayed increased gene expression for chemokines (i.e., CCL2, CCL22, CCL4, and IL8) and colony-stimulating factor (i.e., CSF1) and costimulation of cellular molecules (i.e., CD1B, CD40, and CD209), while expression was diminished for Fc*γ*R III (CD16), CD14, and HLA-DR. Moreover, the Mo(cIgG) had upregulated cytokine genes, including TNFSF14 (LIGHT) and SPP1 (osteopontin), which promote osteoclastogenesis and bone resorption in RA [34, 35]. Flow cytometry confirmed the increased CD40, CD1b, and CD209 expression and the decreased CD16 and CD14 expression (Figure 3(e)). These unique features may provide an opportunity for examining the molecular mechanism of immunity trained by RA autoantibodies.

### 3.3. Comparative Transcriptomic Analysis of cIgG- and *β*-Glucan-Trained Immunity

**Figure 4:**
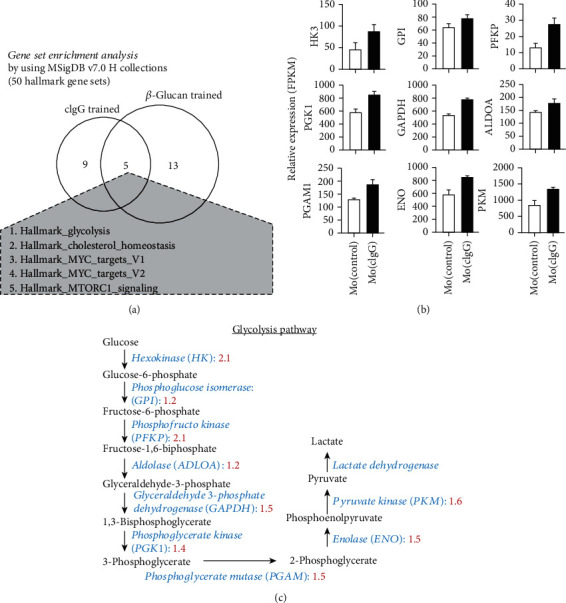
Comparative transcriptomic analysis between cIgG- and *β*-glucan-trained monocytes. (a) Venn diagram of GSEA of trained monocytes using hallmark gene sets collected in MSigDB v7.0. The five shared hallmark gene sets were glycolysis, cholesterol homeostasis, MYC-targets_V1, MYC-targets_V2, and MTORC1_signaling. (b) Relative gene expression of glycolytic enzymes (HK3, fold change (fc) = 2.1, *P* = 0.00453; GPI, fc = 1.2, *P* = 1.75*E* − 11; PFKP, fc = 2.1, *P* = 3.07*E* − 09; PGK1, fc = 1.2, *P* = 2.39*E* − 09; GAPDH, fc = 1.5, *P* = 0; ALDOA, fc = 1.4, *P* = 2.16*E* − 11; PGAM1, fc = 1.5, *P* = 7.34*E* − 09; ENO, fc = 1.5, *P* = 0.00442; and PKM, fc = 1.6, *P* = 2.86*E* − 10) was defined by FPKM (fragments per kilobase of exon model per million reads mapped) values from RNA sequencing (GSE102728) as compared between the Mo(cIgG) and Mo(control). Data (mean ± SEM) are from three independent experiments. *P* values indicate the posterior probability of differential expression (PPDE). (c) Glycolysis pathway from glucose to lactate. Fold changes in glycolytic enzymes were compared between cIgG-trained and control cells, as labeled in red.

As there was high similarity in the trained features observed between the cIgG- and *β*-glucan-primed monocytes, we wanted to know the core regulatory mechanisms for the training process. Therefore, we performed comparative transcriptomic analysis between the cIgG- and *β*-glucan-trained monocytes using GSEA on a hallmark gene set collection (*n* = 50). Surprisingly, there were five shared gene sets: glycolysis, cholesterol homeostasis, MYC-targets_V1, MYC-targets_V2, and MTORC1_signaling (Figure 4(a)). It is widely known that cellular metabolic shift towards glycolysis is a key mechanism in *β*-glucan-trained monocytes, which previous studies have demonstrated extensively [2, 29]. Correspondingly, several rate-limiting glycolytic enzymes were upregulated in the Mo(cIgG) as compared to the Mo(control), including HK3, phosphoglucose isomerase (GPI), phosphofructokinase (PFKP), aldolase (ALDOA), glyceraldehyde-3-phosphate dehydrogenase (GAPDH), phosphoglycerate kinase (PGK1), phosphoglycerate mutase (PGAM1), enolase (ENO), and PKM (Figures 4(b) and 4(c)). These findings suggest a role of metabolic shift towards glycolysis in the formation and maintenance of trained immunity.

### 3.4. cIgG-Trained Signatures Were Enriched in RA Synovial Tissues

**Figure 5:**
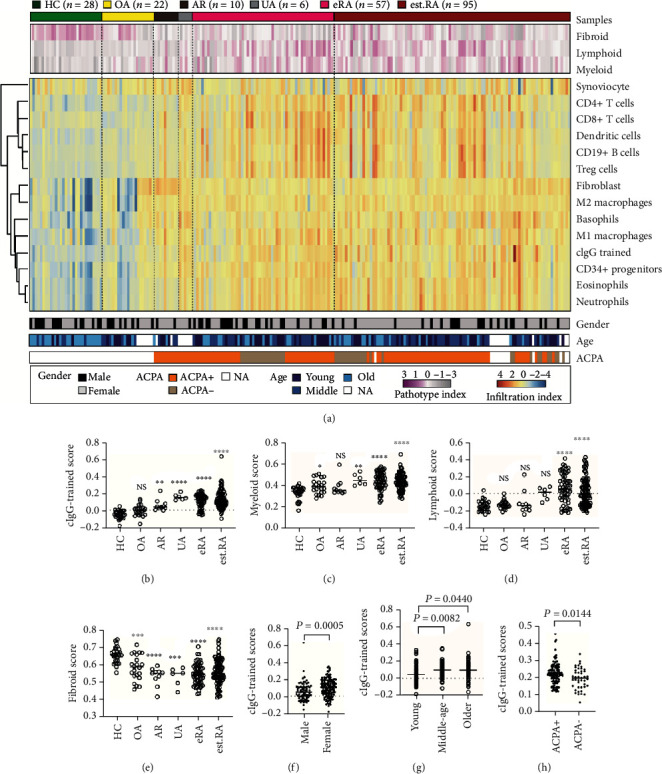
cIgG-trained signatures enriched in synovial biopsies. (a) Heatmap showing hierarchical clustering according to disease status, pathotype, cell-specific gene score, and clinical information. The synovial samples were from healthy donors (HC, *n* = 28) and from patients with osteoarthritis (OA, *n* = 22), arthralgia (AR, *n* = 10), undifferentiated arthritis (UA, *n* = 6), early RA (eRA, *n* = 57), and established RA (est.RA, *n* = 95). (b–e) Enriched scores of cIgG-trained monocytes and three pathotypes among the groups. Comparison of cIgG-trained scores in groups according to (f) sex, (g) age, and (h) ACPA status is shown. Each dot represents one donor. NS: no statistical significance; ∗*P* < 0.05, ∗∗*P* < 0.01, and ∗∗∗*P* < 0.001. The Kruskal–Wallis test, followed by post hoc testing by Dunn's multiple comparison test, was used for comparing >2 groups. The Mann–Whitney *U* test was used for comparing two groups.

Although the molecular mechanism of trained immunity has been studied intensively [2, 3, 36], the underlying role in vivo remains largely unknown. Recent studies [10, 11, 30] have described the presence of tissue heterogeneity in synovial tissue according to three major histological pathotypes: fibroid, myeloid, and lymphoid. The fibroid pathotype indicates a fibroblastic pauci-immune pathotype, while the myeloid pathotype indicates myeloid lineage predominance but a paucity of B cells/plasma cells and the lymphoid pathotype is dominated by lymphoid lineage infiltration (T cells, B cells, and plasma cells) in addition to myeloid cells [10]. Therefore, to gain insight into trained immunity with tissue heterogeneity, we defined the top 100 upregulated genes in the Mo(cIgG) as cIgG-trained signatures via RNA sequencing (Figure 3(a) and Supplementary Table 2) and ran the three pathotype signatures in GSEA against synovial tissues [27] from healthy individuals (*n* = 28) and patients with osteoarthritis (*n* = 22), ACPA-positive arthralgia (*n* = 10), undifferentiated arthritis (*n* = 6), early RA (*n* = 57), and established RA (*n* = 95) (Figure 5(a)). We also analyzed 13 types of immune cells and tissue resident cell populations using their specific gene signatures (Supplementary Table 2).

In comparison with the healthy synovial biopsies, the cIgG-trained signatures were enriched in the preclinical stages of RA from arthralgia and undifferentiated arthritis to early RA, but not in osteoarthritis (Figure 5(b)). Notably, the patients with arthralgia in the present cohort were double positive for ACPA and RF and were at high risk for quickly developing RA [21]. Similarly, the myeloid score was present for the synovial tissues from undifferentiated arthritis to early RA, but not for that from arthralgia (Figure 5(c)). Interestingly, the slight increase in myeloid scores for patients with osteoarthritis indicated that myeloid lineage cells may also play a role in osteoarthritis. Conversely, the lymphoid signature was selectively enriched in early RA (treatment naïve) and established RA (Figure 5(d)), which is consistent with the observation of profound inflammatory response and lymphocyte proliferation. Strikingly, the dramatic decrease in fibroid scores in the disease groups indicated that it is a potential biomarker for health (Figure 5(e)). More importantly, this difference was also sex- and age-dependent, suggesting that women and older people are at high risk for developing RA (Figures 5(f) and 5(g)). In addition, the cIgG-trained scores showed higher enrichment in the ACPA-positive population, while there were no significant differences between the myeloid and fibroid pathotypes (Figure 5(h)).

**Figure 6:**
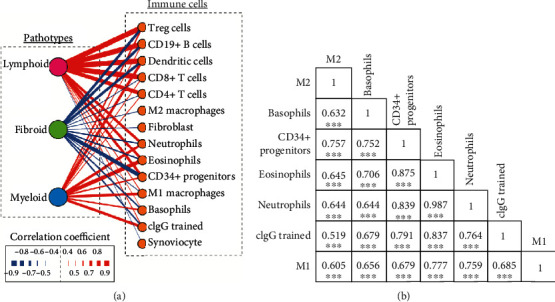
Correlation of RA synovial pathotypes with immune cells. (a) The relationship between the three RA synovial pathotypes, i.e., lymphoid, fibroid, and myeloid, and immune cells, represented by nodes. The interaction profile is shown in the links (lines) between the nodes, and the strength is highlighted by the thickness of the lines and the color. Red and blue lines indicate positive and negative correlations, respectively. (b) Correlation matrix for M1, M2, cIgG-trained monocytes, neutrophils, eosinophils, basophils, and CD34+ progenitors. Pearson correlation coefficients are indicated. ∗∗∗*P* < 0.001.

In the synovial lesion, the infiltrated immune cells correlated highly with the lineage-specific pathotype and correlated negatively with the fibroid pathotype (Figure 6(a)). Importantly, the eosinophil and neutrophil enrichment scores correlated with the lymphoid and myeloid pathotypes, suggesting that these cell populations might be connected by the antigen-presenting cells (APC), leading to lymphocyte and neutrophil infiltration and activation. Strikingly, neutrophils were strongly correlated with cIgG-trained macrophages (Pearson *r* = 0.764, *P* < 0.0001) and less correlated with M1 (Pearson *r* = 0.759, *P* < 0.0001) and M2 macrophages (Pearson *r* = 0.644, *P* < 0.0001) (Figure 6(b)). The cIgG-trained macrophages also correlated strongly with basophils (Pearson *r* = 0.679, *P* < 0.0001) and eosinophils (Pearson *r* = 0.837, *P* < 0.0001). The trained cytokine and chemokine production might help recruit more inflammatory effectors to the inflamed niche, resulting in an amplified inflammatory response, thereby creating a vicious cycle promoting the perpetuation of inflammation. The cIgG-trained macrophages were also associated with CD34+ progenitors (Pearson *r* = 0.791, *P* < 0.0001) (Figure 6(b)), which involves a premature aging process [37], including early events in the shaping of the immune system.

### 3.5. cIgG-Trained Signatures Could Predict the Biological Therapeutic Benefits in RA

**Figure 7:**
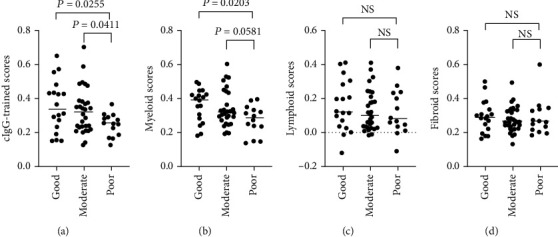
cIgG-trained signatures correlate with clinical response to infliximab therapy. Analysis of synovial tissue transcriptome from 62 RA patients in GSE21537 before the initiation of infliximab (anti-TNF*α* treatment). The clinical outcome by infliximab at 16 weeks was defined by EULAR response criteria. Scores versus EULAR responses are plotted for (a) cIgG-trained scores and the synovial (b) myeloid, (c) lymphoid, and (d) fibroid pathotypes. The Kruskal–Wallis test was performed, followed by post hoc testing by Dunn's multiple comparison test.

Increased TNF*α* production characterizes the predominant feature of trained immunity and has been proven to be the most effective therapeutic target. Hence, we hypothesized that these trained signatures may correlate with clinical response to anti-TNF*α* therapy. Therefore, we used an independent RA synovial gene expression cohort (GSE21537) [38] to validate the baseline cIgG-trained signatures for predicting the clinical response to infliximab against TNF*α* mainly produced by synovial macrophages. Patients were grouped according to their European League Against Rheumatism (EULAR) clinical response to infliximab based on disease remission, monitored by DAS28 (Disease Activity Score including a 28-joint count) at 16 weeks. Remarkably, the baseline cIgG-trained signature expression was significantly increased in patients with good (*P* = 0.0255, Figure 7(a)) or moderate EULAR response (*P* = 0.0411, Figure 7(a)) compared to that of poor responders. A similar correlation was observed between the myeloid signatures with clinical therapeutic response (Figure 7(b)). However, both the lymphoid and fibroid scores, despite also marking the inflammatory process, were not associated with clinical outcomes (Figures 7(c) and 7(d)). The data indicate that cIgG-trained signatures can be used for predicting clinical therapeutic responders to anti-TNF*α* therapy.

## 4. Discussion

Trained innate immunity was first described via exposure to *C. albicans* or *β*-glucan with host protection for reinfection, while the inappropriate activation of trained immunity may cause harmful effects, particularly in inflammatory and autoinflammatory diseases [1, 2, 5, 6]. In the present study, we described how RA-specific autoantibodies could be used for training human monocytes to demonstrate increased responsiveness (Figures 2(e)–2(h)), which reinforces the importance of autoantibody-trained immunity in the pathogenesis of RA. Our results show that the deposits of antibodies against ACPA (predominantly IgG1) and RF (predominantly IgM) exhibited distinct trained properties, which was mainly due to the isotype and its functions. In general, RF IgM and ACPA IgG are the two most remarkable autoantibodies in RA, being present before the onset of disease symptoms by several years, with different clinical information [39, 40]. The fluctuations in RF levels are regarded as inflammatory markers of disease activity and response to therapy. A pathological view of these autoantibodies shows that IgG may activate macrophages through Fc*γ*R engagement, while IgM can induce complement activation [41]. Herein, we observed a trained ability with ACPA IgG, which reflected a distinct function with RF IgM (Figures 2(e)–2(h)). A recent study has suggested that ACPA IgG triggers the amplifying effect of RF IgM on TNF*α* production on human monocytes [42]. This is also in line with previous findings that RA patients positive for ACPA IgG have milder disease activity, while patients who are double positive for ACPA IgG and RF IgM exhibit elevated acute-phase reactants [43, 44]. In contrast to RF IgM, ACPA IgG appears less informative in terms of the association with the severity of radiographic damage in RA [19, 45]. Notably, in terms of cytokine production, the cIgG-trained monocytes were relatively quiescent. However, the cIgG-trained monocytes could produce chemokines during this process, which may provide strong chemotactic signaling for leukocytes and myeloid lineage cells (Figures 2(g) and 2(h)). In parallel, upregulated CSF1 and receptor activator of nuclear factor-*κ Β* ligand (RANKL) signaling was also seen in cIgG-trained immunity (Figure 3(d)), which may induce osteoclastogenic differentiation that facilitates cartilage damage and bone destruction [46].

Broad screening of the autoantibodies in synovial tissue has revealed abundant deposits of ICs [17], which suggests that using cIgG for mimicking ICs is pathophysiologically relevant. Here, we compared the transcriptomes of human monocytes trained by *β*-glucan and those trained by cIgG and identified five core regulatory pathways, representing glycolysis and the mTOR signaling pathway (Figure 4(a)). This may suggest new therapeutic and preventive strategies for autoimmune diseases by targeting the specific mechanisms of trained immunity, such as glycolysis and the mTOR signaling pathway. In addition, there are many similarities between RA synovial macrophages and trained immunity. For example, Ciurtin et al. used proton magnetic resonance spectroscopy (MRS) and reported elevated lactate and diminished glucose concentration in synovial fluids, which suggested increased glycolytic activity in synovia [47]. In the present study, several rate-limiting glycolytic enzymes (i.e., HK3, PFKP, ENO, and PKM2) were upregulated in the cIgG-trained monocytes, which has also been demonstrated in RA inflammatory macrophages [14, 15]. Moreover, the Akt-mTOR-HIF1*α* signaling pathway-dependent induction of aerobic glycolysis represents a metabolic basis for trained immunity [3]. The inhibition of mTOR reduced synovial osteoclast formation and protected against local bone erosion and cartilage loss [48]. In addition, epigenetic modification also appears to be a crucial mechanism for trained immunity [2]. Interestingly, our recent work [16] defined significant histone modifications by H3K4 trimethylation on various inflammatory cytokines in cIgG-trained monocytes, which provided an epigenetic memory for long-lasting capacity without genetic changes. Treatment using TNF*α* inhibitors (etanercept and adalimumab) could downregulate H3K4, H3K27, H3K36, and H3K79 trimethylation at the promoter site of CCL2 (MCP-1) in monocytes, which correlated with RA disease activity [13]. Therefore, the implications of trained immunity may suggest novel therapeutic and preventive strategies for RA by targeting the molecular mechanisms of trained immunity, including immunological and metabolic to epigenetic approaches.

There is increasing recognition of the importance of synovial tissue as a primary pathological site of RA, which is more informative than peripheral blood for the disease. In addition, GSEA can determine the abundance of specific gene sets and has been widely used for investigating immune cell infiltration in the tissue. To further identify the role of cIgG-trained immunity in synovial tissue, we performed GSEA using the top 100 upregulated DEGs of cIgG-trained monocytes against transcriptomic datasets from synovial tissues. The cIgG signatures were strongly enriched in synovial tissue from patients with arthralgia, undifferentiated arthritis, early RA, and established RA, suggesting a historical priming event (Figures 5(a) and 5(b)). It is worth noting that the patients with arthralgia enrolled in our cohort were serologically positive for ACPA and RF at the first clinic assessment and were at high risk for developing RA quickly (Supplementary Table 1) [21]. Therefore, our finding suggests that trained immunity may act as a key inflammatory event at an early stage, which is similar to the findings on chronic inflammatory disorders, such as hyperuricemia and atherosclerosis [5, 6]. Strikingly, comparison of the clinical parameters with the cIgG-trained signature indicated that the high-risk factors for RA are female sex, older age, and ACPA+ status (Figures 5(f)–5(h)), which is consistent with the findings of epidemiological studies.

Recent studies [10, 11, 30] have described the presence of tissue heterogeneity in synovial tissue in the form of three major histological pathotypes: fibroid, myeloid, and lymphoid. Importantly, the infiltrated myeloid lineage cells are a critical driver of pathogenesis in early RA [10, 11]. To address the role of trained immunity in vivo, the gene signatures from cIgG training were compared with the three pathotypes and showed higher similarity with the myeloid pathotype than with M1 or M2 macrophages. The fibroid pathotype was clearly abundant in healthy tissues (Figure 6(a)), suggesting a defined endotype but not end-stage disease characteristics. However, the lymphoid pathotype has been strongly correlated with disease activity, acute-phase reactants, and disease-modifying antirheumatic drug (DMARD) response at 6 months [10]. Furthermore, we constructed a correlation matrix of infiltrated immune cells (Figure 6). Strikingly, the cIgG training was strongly correlated with infiltrated leukocytes, represented as neutrophils, basophils, eosinophil, and CD34+ progenitors, which suggests a potential role for trained immunity in shifting immune cell infiltration. In addition, the baseline of the cIgG-trained signatures was significantly increased in patients with good or moderate EULAR response (Figure 7(a)) as compared to that of poor responders. The data indicate that cIgG-trained signatures can be used for predicting the clinical response to anti-TNF*α* therapy.

## 5. Conclusions

Our results indicate that RA-specific autoantibodies can induce trained immunity in the inflamed lesion, suggesting a historical priming event in the pathogenic process. In addition, the expression of cIgG-trained signatures in synovial tissues played a predictive role in the response to clinical therapy. Therefore, our study may not only advance the understanding of RA pathogenesis but may also suggest novel therapeutic and/or preventive strategies for autoimmune diseases.

## Data Availability

The data that support the finding of this study is available on request from the corresponding author.

## Supplementary Materials

Supplementary Materials Supplementary Table 1: information of patients. Supplementary Table 2: gene lists used in this study.
