# Supplementary material for: Disease-Specific Autoantibodies Induce Trained Immunity in RA Synovial Tissues and Its Gene Signature Correlates with the Response to Clinical Therapy
Source: Mediators Inflamm. 2020 Oct 6;2020:2109325. doi: 10.1155/2020/2109325 (PMC7558774; doi:10.1155/2020/2109325)
Supplement: Supplementary Materials — Supplementary Table 1: information of patients. Supplementary Table 2: gene lists used in this study. [file 2109325.f1.zip › Supplementary Table 2.docx]

| **Supplementary Table 2 Gene lists used in this study** | | | | |
| --- | --- | --- | --- | --- |
| **Name** | **Source** | **Reference** | **Genes** |  |
| Basophils | FANTOM5 | Lewis, M.J.*et.al.****Cell reports***, 2019 | IL4/THAP9/WRAP53/LOC401588/DDIT3/HRH4/IFNG/TRDMT1/EAF1/HSPC081/KLF6/LOC644727/AKIRIN2/DUSP10/RNU6ATAC/RASGEF1B/MANBAL/HSPA5/XCL1/ |  |
| CD19+ B Cells | FANTOM5 | Lewis, M.J.*et.al.****Cell reports***, 2019 | FCER2/PNOC/CD79B/IGHM/TCL1A/LOC283663/IGHD/FCRL1/FCRL3/E2F5/FAM129C/WDR11/KIAA0226L/IL4R/HLA-DPB1/BANK1/MS4A1/BTLA/TMEM156/TCL1B/CD79A/FCRL2/RALGPS2/CPNE5/HTR3A/CD72/CD40/CLECL1/HLA-DRA/BLK/HLA-DOB/CD22/CXCR5/CLEC17A/LOC646626/CD19/ZNF860/VPREB3/TNFRSF13C/TLR10/IGLL5/ZBTB32/TLK2/WDFY4/POU2AF1/LCN10/HLA-DOA/RFX5/CIITA/FCRLA/P2RX5/LOC400958/PAX5/BACH2/LINC00494/TNFRSF13B/FCRL5/SPIB/C12orf74/FOXP1/TAPT1/CD74/TNFRSF17/PLEKHF2/COL19A1/CD180/FAM177B/BLNK/HLA-DMB/PPAPDC1B/STAP1/DUS2L/SNX22/CNR2/MARCH1/USP6NL/SNX2/PTPRCAP/DRAM2/LOC100129196/STX7/SMC6/ |  |
| CD34+ Progenitors | FANTOM5 | Lewis, M.J.*et.al.****Cell reports***, 2019 | KIAA0087/LCOR/DDIT3/LOC284648/ATP2C1/ATP8B4/ARL5B/ANKRD28/FLJ31945/TRIM24/FAM65C/MED12L/SKIL/LOC643936/MAFF/HSPA5/SEPT7L/GRASP/CHST11/USP6NL/IL21R/ |  |
| CD4+ T Cells | FANTOM5 | Lewis, M.J.*et.al.****Cell reports***, 2019 | P2RY8/SNORA16A/SCARNA5/SNORA8/JUN/HARBI1/RCAN3AS/FHIT/LOC100128420/ICOS/C14orf178/SMAD7/CAGE1/LOC100288869/PHOSPHO2/NDNL2/RMRP/SNORD89/HAUS3/FLJ43663/PASK/FLT3LG/SNORA65/LOC730961/PIK3IP1/BTBD9/PBX4/SNORD35B/FBLN7/PER1/C3orf71/ATG16L1/SNORA53/CD6/CD5/HERPUD2/KCNA3/SNORD17/JMY/ZNF329/ZNF335/AXDND1/LOC441461/TNFRSF25/RORA/EML4/CNST/ATXN7/SCARNA12/POLR3E/CCNL1/TNFRSF4/ZBTB1/TBCC/WHAMM/CDC14A/BCL11B/NBPF8,p1/NR1D2/RNF125/ZFP36L2/TOE1/UMODL1/SNHG1/RHOH/HECA/KIF20B/RFX3/ZBTB25/MIR29B1/JOSD1/EIF1AD/SNORA12/PTGER4/ZC3HAV1/ARL4C/DOCK10/USP3/YPEL5/NR4A2/PAPD5/BCAS2/SMG6/USP36/KBTBD2/JUNB/SPOCK2/CDK13/LTB/SRSF7/BTG1/ITPKB/ZNF767/LOC154761/FLJ13224/ZNF295/PBXIP1/SMEK1/ |  |
| Treg cells | FANTOM5 | Lewis, M.J.*et.al.****Cell reports***, 2019 | C15orf53/FOXP3/IL2RA/CCR8/CCR4/CTLA4/CCR6/TBC1D4/CCR10/OR1F2P/CXCR6/STAM/GPR25/ZBTB32/CD28/RCAN3/TIGIT/ICOS/PYHIN1/PMAIP1/LEPROTL1/THEM4/CDKN1B/GPR15/HAUS3/ZNF101/TNFRSF18/LAIR2/EMBP1/SIRPG/CD2/PTPRCAP/ |  |
| CD8+ T Cells | FANTOM5 | Lewis, M.J.*et.al.****Cell reports***, 2019 | ZNF683/GZMK/GZMA/KLRK1/CD8A/GZMH/TTC16/GZMM/PTPN4/SAMD3/LOC100128420/LDLRAP1/APOBEC3G/GIMAP7/FCRL6/NOSIP/GPR171/TC2N/SCARNA17/FAM113B/ |  |
| Dendritic Cells | FANTOM5 | Lewis, M.J.*et.al.****Cell reports***, 2019 | CLEC4C/LILRA4/PHEX/SIDT1/PPM1J/LRRC36/SYCP2L/PLD4/IRF7/IRF4/IL3RA/TCL1A/CCDC50/KIAA1984/PTCRA/GZMB/FAM129C/KIAA0226L/HHAT/SLC9A11/IRF8/KLRF2/FCHSD2/HESX1/FAM160A1/TCL1B/PLEKHD1/SCT/MAP2K6/TRAF4/HTR3A/SHD/ZNF791/WNT10A/CDYL/SLC15A4/N4BP2L1/DERL3/CLP1/LOC285972/CXCR3/P2RY6/WDFY4/TLR7/VASH2/ERP29/GPR114/TTC24/SEC61B/NUDT17/DUSP5/TLR9/P2RY14/C18orf1/TMIGD2/SPIB/TRIT1/KIAA1274/ERN1/CRYM-AS1/IFI44L/BLNK/ZNF852/CCR2/UNC93B1/SPCS1/FLT3/AMIGO3/KCNK17/ALG2/AKAP13/CSF2RB/SRSF6/CLN8/TIFAB/C16orf93/CD2AP/MTMR1/TM9SF2/ZBTB33/MAPKAPK2/ZBTB2/RIOK1/UBE2J1/PARP10/FUT7/THUMPD2/GRASP/ST3GAL2// |  |
| Eosinophils | FANTOM5 | Lewis, M.J.*et.al.****Cell reports***, 2019 | ARFIP1/BCL2A1/FBRS/PPCDC/SLC12A6/FLJ22447/VNN3/NBN/PELI1/ADORA2A/IL1R2/NEDD9/TMCC3/GP5/ATG2A/C2CD3/PRKCH/OSM/LOC257358/IL8/CCDC147/SOS2/MAPK13/TREM1/MIR3945/SYAP1/AQP9/PDE4B/N4BP1/LMNB1/MIR4736/LINC00528/DYSF/AMPD2/AP3S2/SRGN/C5AR1/GPR97/ZCCHC6/TMCC1/IVNS1ABP/DOCK4/CASS4/PHF20L1/C10orf46/IRAK2/IFITM2/LOC646471/LOC100289495/IPMK/G0S2/SIPA1L1/PLAUR/TXNRD1/NINJ1/ACAP2/RASSF5/C17orf107/CLEC4E/IFNGR1/HIVEP1/LITAF/PLXNC1/GNAI3/DENND5A/GGA1/KIAA0232/THAP9/TMEM127/ALOX5AP/LIMK2/SLC9A8/SAMSN1/BIN3/PCNX/CIR1/ZBTB7B/IL4R/LINC00173/FOLR3/KIAA0226L/ZNF668/UBE2R2/NSMAF/GLT1D1/GBP1/ATP11B/SEC14L1/RAB8B/RNF44/ABHD2/ZNF281/CTBS/VNN2/VCPIP1/GAB2/NAMPT/IL1RN/SPAG9/LOC100130597/LCP2/MXD1/TRPM6/PPP2R5C/FLCN/EGR3/TLR1/DOCK5/NEDD8/MIDN/SUMO1P1/GMPR2/LOC100128429/LOC100302650/GPSM3/NFKBIZ/RAB5A/BCL3/LOC401588/B3GNT5/UBE2D3/SLC45A4/FPR2/ZDHHC18/SPATA2L/FLJ36644/EHD1/STK40/FCAR/AHCTF1/KIAA0247/FAM49B/PPP4R2/SLA/LOC100132344/BOD1L/FRMD4B/C12orf35/FBXL13/TLE3/RNF19B/EIF2C3/SH2D3C/EMR3/PNPLA8/XPO6/SHOC2/NLRP6/VPS13B/ABHD13/KCMF1/IFIT3/PREX1/MME/KLHL2/C1orf138/FCGR2A/TMEM154/CDADC1/TREML4/GMIP/MBOAT7/RILPL2/PHACTR1/C9orf72/CSF3R/C15orf29/PLEK/OXSR1/TRIB1/PXT1/LRRC4/SPAG1/SOD2/GPR27/CHST15/CSAD/NFKBIE/CREBBP/JMJD6/LOC648987/FFAR2/IRAK3/CSNK1D/PURB/NFKB2/RNF169/PATL1/KIAA1257/RIT1/ALPK1/ELL/GPR65/CD82/STX3/DIRC2/IFIH1/IRS2/PTEN/PRKD2/GNG2/ZMYND15/B3GNTL1/LYN/AP1G1/OBFC2A/BNIP2/TLR2/ADAM8/DDIT3/ITPRIP/NFKBIA/GCA/TFIP11/IL12B/SNRK/DENND3/GPCPD1/PRR14/GTPBP1/TICAM1/FPR1/C15orf39/BAZ1A/CCDC71L/CEACAM3/REPS2/SH2B2/IL13RA1/TBC1D7/SLC15A4/BRAF/CYB5R4/ESCO1/FLOT1/CXCL16/PIK3R5/SEMA4A/RASSF2/LOC731223/LOC100506801/ANKRD13A/ISG20/RAD18/CFLAR/MIR2110/NRBF2/PLK3/DDX3Y/CEBPB/ICAM1/ZNF267/SAP130/CHMP4B/CCDC134/TREML2/KRAS/C1orf124/BTG2/TMEM167B/SMAP2/ITM2B/TMEM71/ORAI2/ARMC5/TMEM140/TBK1/SMCHD1/PTPRE/TANK/ACSL1/SFT2D1/SSH2/ARL5B/PACSIN2/HNRNPH2/ELF2/SLC22A18AS/UBR2/RNF149/PGS1/ZFAND3/IFRD1/SLC11A1/FNIP1/MON1B/LOC285758/KDM3A/USP4/MBD4/KIAA0317/TSC22D2/MPPE1/PFKFB3/ABTB1/FGD4/CSNK1G2/TAF7/RICTOR/MX2/SLC2A3/ATP2B1/ARHGAP26/C3AR1/RAB21/RBM23/RLF/MAP3K2/FAM8A1/SMPD2/NBEAL2/PNRC1/AGTPBP1/PMAIP1/C12orf61/AFF4/THAP2/LST1/CD83/UBAP1/PHF20/CDKN2D/LOC100287559/PIGV/SP110/TAGAP/NUMB/ZNF107/PIK3CA/MAPK14/ZC3H12A/NUP98/GNA13/LINC00239/FAM160A2/EXOC8/RAF1/TOR1B/HBP1/SRPK1/UHRF1BP1L/TNIP1/LOC100288432/ROCK1/VASP/ABHD5/MSL1/CHD2/ZNF687/DOT1L/SNX18/ZNF81/DSE/MCL1/NR6A1/CHUK/RNF144B/USP15/VMP1/CD55/HSPC081/CYP1B1-AS1/SLC22A4/GALC/SMAD7/CEACAM4/PLDN/STAT5B/TBL1X/CCNG2/STX6/IL1RAP/BRD4/PTAFR/VAV1/LIPN/SCLT1/CHD4/RAB24/PRKRIR/FMR1/FMNL1/RGS2/PLEKHO2/TNFSF14/PTGS2/GIT2/SPATA5L1/SDCBP/MAEA/GBP2/C5orf41/ETF1/CEP63/OASL/FAM53C/DDX3X/NADK/SYF2/RNF31/FGD3/SBNO2/ZFY/LRMP/NFKB1/CSF2RB/CPD/LOC100130027/MEPCE/HERC5/STXBP3/MEFV/FAM49A/EMR2/TLR8/CWC25/MTMR14/PAN3/SKIL/SAT1/ADAM17/LOC646329/SETX/LRRK2/PPIF/RIPK1/SLC6A6/TNFAIP2/RIC8A/TRIM39/STX11/SKIV2L/IFIT2/BLOC1S2/ZNF467/ERN1/YTHDF3/LBR/NFIL3/AFTPH/METTL21A/ARHGAP9/LOC730961/POGZ/ANKRD12/NFKBID/PANX2/RIPK2/CTDP1/LOC728445/EFCAB4B/CD58/ATG7/SPTY2D1/DHX8/PPP1R15A/ZFP91/LCP1/LOC100216545/GK/FAM129A/ICAM3/KPNA4/CAMK1D/ZHX2/TOPORS/NR4A3/TET2/SYNJ1/CXorf21/HAUS3/NR1H2/OSGIN2/UBXN2B/KIAA2018/RB1CC1/PTPN12/JMJD1C/CHIC2/DEDD2/TMEM170A/GRASPOS/INPP5K/MIER1/STK16/GTDC1/BCL10/AKIRIN1/OSBPL11/FGGY/WAPAL/FBXL3/LIN54/C20orf24/YWHAZ/OSBPL2/MIR4745/MED12/RNF103/MIR4530/SH3BP1/TNFAIP3/CREM/CSRNP1/TNFRSF1B/RALGAPA2/DKFZP686I15217/CNOT10/BRD2/EAPP/CCRL2/MIR22HG/BID/MKNK1/CBL/MYO1F/RBM47/CXCR4/WTAP/NFAM1/ATHL1/PEX16/IFITM1/OTUD5/TOM1/MLL3/LOC730227/ZNF263/HOTAIRM1/PSEN1/SPOPL/RANBP2/ZFC3H1/MAPKAPK2/MAP3K8/ZNF292/LYST/COQ10B/ST3GAL2/TINF2/FGR/C1orf55/ARID4B/LOC100131691/LOC389634/ITGAX/C16orf72/IL21R/PPP2CA/VDR/CLEC7A/HCK/FAM214B/NCOA2/IFNGR2/VTRNA1-1/DHX40/WAS/APBB3/GADD45B/ACTR2/C20orf197/PILRA/DAZAP2/RABGEF1/TLR4/PPP4R1/WAC/SLCO3A1/SPATA13/MAFF/GGNBP2/RPGR/NCF2/SIGLEC10/KDM5A/OSBPL8// |  |
| Fibroblast - skin | FANTOM5 | Lewis, M.J.*et.al.****Cell reports***, 2019 | CCDC99/NDUFAF2/C11orf51/GGPS1/UBE2L3/NSA2/DRG1/XRCC6/CETN2/HAT1/MAGOHB/THOC7/ZBTB8OS/PSMC2/DDAH1/UBA3/C6orf62/TTC1/PSMD10/KRT34/SKA2/EIF4E2/BLOC1S3/LLPH/FCF1/SUB1/SRP9/VTA1/FAM114A2/N6AMT2/MOCS2/DNAJC8/C20orf43/UBLCP1/SF3A3/TARDBP/C22orf28/TAF13/CKAP5/PDCL3/MAGOH/KRTAP1-1/NDUFS5/CASP3/SPA17/RPF2/LOC96610/FAM192A/TCEANC2/TXLNA/LARP7/SSB/LEO1/DCAF13/CHMP5/MSL3P1/PSMA4/VDAC3/SLU7/PEX2/C1D/HYLS1/SHFM1/ANP32E/PRPS1/KIF18A/MYL12A/VPS26A/ENY2/PSMC3IP/CPSF2/BLZF1/RWDD2B/BUD31/PFDN4/MFAP1/CLOCK/C11orf58/SMU1/JRKL/TCEAL8/UTP11L/LSM3/PUS10/SNUPN/HNRNPR/PHB/PMM2/RABL3/TATDN1/CCDC90B/DNAJC24/UFM1/TAX1BP1/C11orf57/GTF3C6/ERGIC2/UBE2K/C11orf74/DPM1/CCDC36/CCDC25/ZCCHC17/RFC1/RAD21/TPI1/SNRPG/CRYZL1/CDC23/RRP15/GLOD4/TIPIN/HNRNPA2B1/API5/FAM98A/ATP5H/CWC15/TARS/SRP14/RPL7/SF3B14/HBS1L/COX16/DDX46/EFTUD2/LIN7C/GTF2H3/NUP188/YAE1D1/IER3IP1/HNRNPH1/ZCCHC9/EIF2S1/SMARCE1/PPIL3/DIEXF/PSMD4/44076/FAM175B/TMEM242/MTAP/CINP/BRCC3/PARK7/PDCD10/COPS2/TAF9/VAMP3/BZW1/NXT2/SNX7/PSMC5/POLR2K/C6orf211/METTL14/C2orf18/TTC37/ESF1/GTF3C3/CDC5L/PRIM2/ZNF146/RHOA/TFB2M/PSMC1/RPS26/RACGAP1/NARS/ZFP161/BTF3L4/PCNP/CAPZA1/PSMA1/METTL13/GABPB2/YWHAE/ANAPC13/GMFB/HMGB3/UBE2V1/SUMO1P3/PPP1R9B/LUZP6/C12orf48/SNX11/TIPRL/RARS/NCL/HAUS8/RPL14/ZWILCH/TXNDC9/SNRPD3/ZNF215/MFN1/BRD8/POMP/WDHD1/RAD17/NFYB/STX8/CFL2/NOL11/COPZ1/MRPL51/SARNP/ACTL6A/DLD/KIAA0020/PIGK/TBCE/C5orf51/LYSMD3/WDR3/LSM5/CENPE/XRCC4/TSPAN31/SUMO1/NVL/DENR/UAP1/LARS/FOPNL/RBM41/CHCHD7/GEMIN6/IPO8/MMADHC/RSL24D1/MMP3/ASNSD1/PIP5K1A/UBAP2L/TBC1D23/SAR1B/VPS29/SSR1/LSM7/PSMB4/MYCBP/PSMA3/SAR1A/MICU1/PHAX/MNAT1/MRPL42/TRNT1/LYRM2/FAM204A/OLA1/SUPT6H/APTX/NASP/CXorf26/CEP152/NRD1/PIN4/RBBP8/ERLIN2/MRPS33/SEC11A/CEP57L1/CWC27/BFAR/ST13/NMD3/NHP2L1/TTK/ECI2/ATXN3/MRPL40/EXOSC2/ANXA1/CCDC75/COPS4/DDX18/CENPN/FANCB/CSDE1/CENPK/FKBP3/UMPS/PBK/EAPP/PSMA5/GAR1/CBX5/RIOK2/HMGB1/SH2D4A/XRCC5/MRPL1/TPMT/CCDC58/YWHAZ/RBM8A/UBE2V2/SNORD97/RPL36AL/CDK7/TYW3/VRK1/CENPQ/ECT2/PSMC3/FAM36A/TAF15/CCT8/NCAPG2/TMSB10/SLIRP/TRIAP1/CYB5B/NSMCE1/SSBP1/MKI67IP/NOL10/HNRNPC/MCTS1/SSX2IP/TIMMDC1/ALDH7A1/C15orf63/MORF4L2/NUSAP1/RAD51AP1/EIF4B/TIMM8A/SUMO2/ABCE1/SENP3/NUP43/PSMD14/MRPS18C/ZRANB2/ERH/RAD51C/PPIE/MLH1/TWF1/PRC1/NUDCD1/TIMM21/FAM54A/NAP1L4/GIN1/CDC26/THUMPD3/BPNT1/RNF34/RTCD1/TRAPPC4/PMS1/SUGP1/PLRG1// |  |
| Neutrophils | FANTOM5 | Lewis, M.J.*et.al.****Cell reports***, 2019 | ARFIP1/DOK3/ENTPD1/PROK2/VNN3/C2CD3/PELI1/SLC12A6/FLJ22447/PPCDC/ATG2A/TMCC3/FBRS/BCL2A1/IL1R2/NEDD9/ADORA2A/NBN/IL18RAP/IVNS1ABP/KDM6B/DYSF/MIR4736/CASS4/TMCC1/IL8/AMPD2/C10orf46/LOXHD1/DOCK4/PHF20L1/LMNB1/C5AR1/ZCCHC6/PDE4B/MIR3688-2/N4BP1/LINC00528/AP3S2/GPR97/SYAP1/TREM1/AQP9/MIR3945/MAPK13/SOS2/CCDC147/SRGN/GDAP2/MEGF9/CLEC4D/ZBTB7B/IPMK/PCNX/C20orf43/UBE2R2/UBN1/ATP11B/PYGL/GNAI3/SAMSN1/IL4R/CTBS/C17orf107/CLEC4E/BIN3/RASSF5/GLT1D1/TXNRD1/PLXNC1/IFITM2/RNF44/ZNF281/RAB8B/LITAF/ABHD2/LINC00173/KIAA0232/TMEM127/IFNGR1/AGPAT9/NSMAF/LIMK2/ALOX5AP/ACAP2/SEC14L1/DENND5A/NINJ1/SLC9A8/G0S2/ZNF668/ARHGAP30/PLAUR/CIR1/SIPA1L1/ABHD16A/LCP2/KCNJ15/VNN2/MMP25/TRPM6/BOD1L/NAMPT/RAB27A/C1orf138/XKR8/NLRP6/FCGR2A/GMPR2/TMEM154/FBXL13/EMR3/MIDN/CR1/FLJ36644/TLR1/SH2D3C/B3GNT5/GPSM3/XPO6/ZDHHC18/VPS13B/LOC100130597/MME/FRMD4B/VCPIP1/SLC45A4/SLU7/NFKBIZ/SUMO1P1/GAB2/C12orf35/TLE3/SLA/FCGR3B/LOC100130442/FAM49B/PREX1/BCL3/ABHD13/PNPLA8/LOC100132344/RNF19B/KCMF1/FCAR/CEP19/CXCR1/STK40/CXCR2/MXD1/LOC100128429/MIR3610/RAB5A/KLHL2/ULK1/DOCK5/EHD1/SPAG9/SHOC2/RAB31/FPR2/KIAA0247/AHCTF1/PPP2R5C/UBE2D3/SNRK/GPCPD1/NFKBIE/FFAR2/PIGX/B3GNTL1/TRIB1/PRR13/REPS2/CSAD/OXSR1/SH2B2/BNIP2/AP1G1/ADAM8/STX3/KIAA1257/SPG11/WIPF1/CYB5R4/PPP1R18/DIRC2/GPR27/SNX13/ALPK1/C15orf39/ITPRIP/SNORD99/GPR65/RGS18/NFKB2/GNG2/RIT1/OBFC2A/CCNJL/GTPBP1/LYN/ZNFX1/CSNK1D/BRAF/IL13RA1/LRRC4/ESCO1/CREBBP/DENND3/CDADC1/SLC15A4/TAX1BP1/GMIP/RNF24/CSF3R/PATL1/C9orf72/MBOAT7/JMJD6/SOD2/ELL/CD82/PRKD2/RNF169/PRR14/PLEK/ZMYND15/CHST15/EIF2C4/ZNF438/PXT1/GCA/ZNF705A/TLR2/NIN/H3F3B/IFIH1/PTEN/STAT6/FPR1/IRS2/CEACAM3/RICTOR/PGS1/PKN2/ABTB1/RASSF2/FYB/CASP5/MNDA/IGF2R/PTPRE/MX2/SMAP2/FNIP1/SLC2A3/UBE2D1/OXER1/LRP10/CEBPB/NRBF2/APOBR/FLOT2/TMEM71/CLP1/ACSL1/UBR2/MBD4/SLC11A1/ANKRD13A/SMCHD1/MIR3064/SSH2/ZFAND3/MIR2110/PIK3R5/TREML2/ARHGAP26/ITM2B/CSNK1G2/ORAI2/TAF7/RNF149/SEMA4A/LOC100506801/USP4/ATP2B1/SCARNA10/PFKFB3/TMEM167B/MON1B/RBM23/CXCL16/WDR26/CCDC134/MYO9B/ZNF267/NLRP12/TBK1/MPPE1/PTBP3/TSC22D2/PLCG2/PLK3/TNFRSF10C/RPGRIP1/PPP6C/ELF2/SAP130/FLOT1/IQGAP2/P2RY13/HNRNPH2/FGD4/TMEM140/KIAA0317/IFRD1/BTG2/CFLAR/LOC731223/APAF1/PACSIN2/PGCP/ZNF107/PHF20/SDCBP/PAPD4/PNRC1/GBP2/TBL1X/ABHD5/GMCL1/GALC/TRAPPC8/GNAI2/CEP63/EXOC8/CDKN2D/TAGAP/BRD4/ROCK1/STAT5B/CEACAM4/ARAP1/C5orf41/RAF1/HBP1/VMP1/SNX18/FMR1/NUP98/LILRA6/PTAFR/SCLT1/C7orf60/RGS2/EP300/MAPK14/UHRF1BP1L/TSHZ3/VASP/RNF144B/SPATA5L1/UNKL/ETF1/FMNL1/MAP3K2/PPP1R9B/MSL1/RLF/CYSLTR1/FAM8A1/MCL1/PPP4R1L/NUMB/IL17RA/SNORD28/GIT2/TNIP1/SP110/USP15/ARHGAP25/IL1RAP/GNA13/MAEA/NBEAL2/CHD2/CHUK/SRPK1/VAV1/FIP1L1/CSF2RA/ZNF687/UBAP1/AFF4/H3F3AP4/LINC00282/CD55/ITCH/CHD4/LRRFIP1/EMR1/PLEKHM1/FAM160A2/CAPZA1/GSK3B/ADAR/PRKRIR/MAP3K5/SLC22A4/FGL2/KDM3B/FAM13B/AGTPBP1/PIK3CA/TPR/LIPN/NR6A1/ITSN2/ITPK1/CPD/ELMO1/KIAA2018/TBC1D23/ARHGAP9/RAB35/CAMK1D/UBXN2B/SAT1/MAP2K3/GTDC1/EVI2B/NADK/CTDSP2/TOPORS/LRRK2/LBR/TLR8/SLC6A6/FGD3/CSF2RB/IFIT2/CHIC2/FAM49A/SNORD1B/LRMP/AKTIP/ADAM17/PHF21A/APBB1IP/ZNF467/DHX34/KIAA1109/DEDD2/AKIRIN2/RBPJ/IGSF6/NFIL3/ICAM3/YTHDF3/STAT3/RB1CC1/TET2/CPPED1/TRIM39/GK/BCL6/JMJD1C/ATG7/MED25/TOX4/AKIRIN1/MIER1/MEFV/STXBP3/SYNJ1/DHX8/EFCAB4B/CWC25/OSBPL11/SETX/CTDP1/NRD1/KAT6A/RAB2A/ZNF552/SPTY2D1/INPP5K/TMF1/SF3A1/SBNO2/PAN3/NUP50/NFKBID/EMR2/CDA/HNRNPK/PANX2/HSD17B11/LPCAT2/NDE1/BCL10/CD58/MLKL/FAM129A/HERC5/POGZ/OSGIN2/USP32/DDX3X/QPCT/MTMR14/ANKRD12/RFWD2/C14orf118/TNFAIP2/LCP1/ERN1/LOC646329/STX11/NMI/UPF2/SKIV2L/NR1H2/FAM53C/ISL2/GMEB2/LPGAT1/SECTM1/STX5/DAPK2/DHRS7/NCOA2/OSBPL2/MTF1/MYO1F/PPP4R1/VPS8/TINF2/GNG10/ITGAX/SPATA13/SPOPL/NFAM1/ATF6B/TSPAN2/C1orf55/BID/LY96/DAZAP2/CAB39/DCAF5/TLR4/UBE2J1/ARID4B/LOC730227/OTUD5/RBM47/SLC15A3/ACTR2/NCF2/PIAS1/ST3GAL2/PPT1/PILRA/OSBPL8/HLX/KDM5A/C16orf72/TNFRSF1B/MGRN1/TBXAS1/CSRNP1/VAPA/COQ10B/SIRPB2/CD97/SLC44A2/FAM214B/MED12/DKFZp761E198/LIN54/APBB3/HERC3/AMICA1/LMBRD1/RPGR/RGS19/LOC100507392/SIGLEC10/ATHL1/FGR/MIR4647/PBX2/NCOA4/RNF103/VDR/FBXO38/CLEC7A/SLCO3A1/CNOT10/RASGRP4/ANP32A/CXCR4/TOM1/ASB7/LRRC25/BRAP/RC3H1/TRIM25/WAS/ZNF292/WAPAL/CCNDBP1/MAP3K8/C20orf197/PSEN1/MKL1/CREB5/MLL3/HNRNPC/SH3BP1/LYST/RALGAPA2/XRN2/DOCK11/SH3GLB1/IFNGR2/SPG21/ZNF263/ACTR3/IFITM1/DHX40/TESK2/SOCS3/C19orf35/FGGY/ALOX15// |  |
| Synoviocyte | FANTOM5 | Lewis, M.J.*et.al.****Cell reports***, 2019 | TSLP/IL26/ACAN/WISP2/LINC00161/FOXS1/TRH/IL13RA2/ANGPTL5/PPAP2B/NTN4/SEMA3C/TNFSF15/ELN// |  |
| cIgG Trained | GSE102728 | Zhong, Q, *et. al.* **JI**, 2018 | IL6/PTGS2/CCL20/TNF/CSF2/IL1A/INHBA/IL1B/IL23A/CCL4/CXCL1/CXCL3/F3/CCL1/IRG1/CCL3L3/CCL3/CCL3L1/CSF3/CCL4L1/OASL/CXCL2/TNFSF15/HCAR2/DNAAF1/MIR155HG/CXCL10/IFIT2/SERPINB2/IL36G/PMAIP1/EDN1/LOC731424/IL8/TNFAIP6/HCAR3/CCL8/GREM1/ELOVL7/IFIT3/ADORA2A/DUSP5/OCSTAMP/GBP5/ITGB3/GAL/MAMLD1/HES4/ITGB8/IL27/CCND1/LIF/FLT1/PTX3/AKAP2/TRAF1/MMP1/TFPI2/LOC646329/ISG15/CRLF2/PELI1/IL36RN/HS3ST3B1/PHLDA2/ISG20/IFI44/ADORA2A-AS1/GCH1/IFIT1/ZG16/EREG/HRH1/ABTB2/TNIP3/CD80/GBP1/ASAP2/HERC5/G0S2/RGS16/CCNA1/CCL22/CCL2/CSF1/MFSD2A/CLCF1/SERPINB9/CFB/GEM/RSAD2/ZC3HAV1/JAG1/STARD4/SPP1/IL1RN/IL7R/IER3/NR4A3/FERMT2// |  |
| M1 macrophage | GSE5099 | Martinez, F.O *et, al.* **JI**, 2006 | ABCB10/ADH5/AIMP2/ALDH16A1/AMPH/ANKS3/API5/ARHGAP24/ASF1B/ATAT1/ATP5MD/BAG3/BCL2A1/BRAP/C12orf57/C18orf21/C1orf158/C1QBP/CADPS/CALCR/CALML4/CCDC77/CCN2/CCR5/CCT7/CDADC1/CDC14A/CDC42BPG/CDK8/CENPJ/CEPT1/CFAP161/CHEK1/CMTM2/COA5/CORO7/COX6A1/CRTAP/CS/CTDSP2/CYB561A3/DBI/DCAF13/DERL2/DHX15/DIMT1/DNMBP/DOK1/DTX2/EHD4/EIF2D/EIF4H/EIF5A/EMP3/ENDOD1/EPHA2/ERGIC3/ETV6/EWSR1/FAM168B/FAM192A/FAM98C/FANCF/FBXW10/FOS/FUS/FYB1/GLRX5/GMFG/GNAS/GSAP/H2AFX/HAGH/HCK/HEMGN/HERPUD1/HEXB/HIGD2A/HLA-B/HLA-C/HNRNPDL/HOXB6/HOXD8/HSBP1/HSPA2/HSPH1/IER2/ILF3/INO80C/ISY1/ITGB1BP1/ITPA/KBTBD2/KHDRBS1/KXD1/LPP-AS2/LRRC8C/LSM2/LY6D/MAGED2/MAP2K7/MARVELD1/MFSD5/MICAL1/MIF/MRPL45/MSTO1/MXD1/NDUFAF6/NEK3/NFKB1/NPM1/NPRL2/NUF2/OTOP1/PABPN1/PANK4/PARK7/PDSS2/PDXK/PHKA1/PIGL/PLBD1/PLXNB2/PPIG/PPP1R3C/PPP4C/PRKAR1A/PROSER1/PRPF38A/PRPF6/PSAP/PSPC1/PTGR1/PYCR3/R3HDM4/RAB1A/RAB43/RACK1/RASL11B/RBAK/RBBP7/RGP1/RGS2/RHBDF2/RIPOR1/RIT1/RNF165/RNF219/RPL24/RPL4/RPL5/RPL7A/RPS4X/RPS8/SCARA5/SFT2D3/SH2B2/SH3RF2/SLC25A3/SLC39A9/SMAD2/SMAP2/SMIM30/SMS/SMYD4/SNRNP70/SPART/SPEF2/SRBD1/SRSF5/STRIP1/STUB1/STX16/SUN3/TBC1D9B/TLE1/TMA7/TMEM107/TMIGD1/TP53RK/TPM3/TRAPPC6A/TRMT2A/TTC21A/TTLL1/TUBA1A/TUBA1B/TXLNB/TXNIP/UBAC1/UBE2V2/UNC5B/VPS35L/WDHD1/WDR1/ZBTB8OS/ZMYND11/ZNF346/ZNRF1/ |  |
| M2 macrophage | GSE5099 | Martinez, F.O, *et, al.* **JI**, 2006 | ABCC12/AC004151.1/AC124319.1/AC129492.1/ACAP3/AGO2/ANKRD37/APOL2/ARL5A/ARMC10/ATP2A2/ATP5F1E/ATP6V1C2/ATP6V1G2/B3GAT1/BET1/BTBD8/C19orf12/C1D/C4orf17/C5orf56/C5orf58/CACNG6/CASC4/CASP9/CCDC88B/CCNA2/CDK5R1/CELF5/CEP104/CHMP5/CKAP2/CLN3/CLPTM1L/CMYA5/CNOT1/CNTNAP1/COMMD7/COPS5/COPS9/CORT/CPT1A/CRKL/CRTC3/CTSZ/CYBC1/DCP2/DNAJB2/DNAJB4/DNAJC21/DSN1/DYNLL1/EGFL6/EGLN1/EIF4G2/ESRP2/EVI2B/EYA3/FAM168A/FAR1/FKBP14/FSD2/GALNT9/GEMIN5/GIMAP2/GMDS/GNG13/GOLPH3/GPHN/GREM2/GRK6/GTF2A2/GTF3C3/HCRTR2/HEMK1/HEXD/HIBCH/HNRNPA2B1/HNRNPC/HSPA5/ICA1/IDUA/IMMP1L/INPP4A/KCNV1/KLHL9/KPNA4/LAT2/LINC00607/LINC00895/LINC01146/LPAR1/LRCH1/LRRC1/LYRM2/MAGI3/MALT1/MAT2B/MCUB/METRN/METTL15/MIR622/MKNK1-AS1/NAT16/NCKIPSD/NFIX/NIN/NPSR1-AS1/NPTN/NUP43/OPA3/OR2B6/OR51B5/OR7D2/OSBPL8/P4HA1/PAH/PAIP1/PAK2/PARP8/PHAX/PHF8/PLEKHS1/PLS3/PNRC2/POMGNT2/POU1F1/POU2F3/PPIL4/PPP1R3C/PRR22/R3HDM2/RAB24/RAB6A/RAET1E/RASL11A/RIMS4/RNF20/RUFY1/SAFB/SEC24A/SERP1/SIRT3/SIX6/SLC26A6/SMAP2/SMIM30/SMURF2/SNX27/SSBP3/STON2/STRAP/STXBP3/STYXL1/SUCLA2/SYN2/SYNJ1/TAF3/TAS2R1/TET3/TFF3/TMA7/TMC7/TMEFF1/TPM3/TRANK1/TRRAP/TUBE1/TULP4/UBE2CP4/UBE2L6/UBR1/VEGFA/WDR33/YME1L1/ZBTB18/ZDHHC20/ZNF137P/ZNF264/ZNF277/ZNF425/ZNF439/ZNF493/ZNF526/ZNF589/ZNF638/ZNF708/ZNF738/ZNFX1// |  |
| Lymphoid | Michgan RA cohort | Dennis, G, et, al. Arthritis Res Ther, 2014 | RLN2/GNGT2/SUB1/CHDH/LRMP/GMIP/LIMD2/KSR2/IPCEF1/KLRD1/LOC440602/TMC8/SIRPG/TNFRSF13C/SFXN2/C7orf29/MAGEB3/ICOS/FUT10/SLAMF6/TAAR5/TLR10/CFLAR-AS1/FCRL1/DEF6/CD96/LINC00593/CXCL13/PLAC8/FKBP11/INPP5D/XCR1/PVRIG/CD2/PRF1/GATA3/RHOH/GPM6A/SLC30A3/ZNF253/MMP1/TBC1D10C/GLB1L3/DNAH8/EMR3/CD7/CCDC127/GPR114/RASGRP1/PIF1/DKFZp434E1119/ARHGAP25/RALGPS2/MYH15/ADAM28/KCNK10/SELL/BCL11B/CALML3/GZMK/FSCB/KRTAP4-5/PIK3R6/LOC339666/CD3E/IGLJ3/SIT1/S100A7A/AACSP1/CLSTN3/EBI3/LINC00582/FANCA/LOC286009/SLC36A1/CD72/SP140/TAS2R10/SLC2A1-AS1/PBOV1/CD244/LOC150622/ITGAL/TIGIT/BCAS4/C14orf176/CCL22/ZNF93/LOC100132249/IL22RA2/LCK/MIAT/CCR6/SPOCK2/CTLA4/FCRL2/ITGA4/VN1R3/PTPRCAP/MS4A1// |  |
| Myeloid | Michgan RA cohort | Dennis, G, et, al.Arthritis Res Ther, 2014 | RAB20/NAGK/SLC4A1AP/GBE1/LRP12/CXorf26/SLC25A13/HOMER3/JOSD1/CSTA/DESI1/EMR2/S100A9/PAG1/AK4/TTYH3/RPS6KA1/C5AR1/CCL8/CTSL1/DFNA5/AQP9/CD86/ZBTB17/RAD51AP1/SLC38A6/CXCL8/FCGR1B/LMNB1/PLEK/RPF2/PGLS/RASAL2/CD300LF/MYO1F/GMPS/CREB3/MAPKAP1/METTL22/LONP1/MTX1/CTSL2/MNDA/CHST15/HPSE/UGCG/NTMT1/STK40/STARD3NL/TMEM51/NEK6/SCAND1/CTSC/SIGLEC7/NCS1/TGIF1/MRPL55/CCR5/MED10/TTLL12/TNFSF13B/LOC100507642/EPSTI1/HK3/UBE2L6/ACSL3/C19orf70/BCKDK/NFKBIE/TRAPPC5/WDFY4/MAPK13/CENPK/FCGR3B/TFRC/CTSH/LILRB1/ITGB2/TRAPPC3/CKS2/TMEM127/NKIRAS2/CTSZ/ACOT13/STAC3/PLOD3/SH3BP2/CXCL3/CCRL2/CERS6/MARK4/PLEKHO2/HCK/TRAPPC12/TSHZ3/LRRC42/TIMM10/CXCL16/RGS19/METRNL/// |  |
| Fibroid | Michgan RA cohort | Dennis, G, et, al. Arthritis Res Ther, 2014 | NOG/CORO6/HOXD9/STARD9/EEF2K/AGAP11/WSCD2/CRTAP/LOC283713/ERCC1/PLP2/HMGB1/CILP/LOC286161/CMPK1/PKD1/SLC17A5/DIP2C/PTMA/WIPF2/GLT25D2/ISLR2/PRELP/SF3B2/EPS8L2/HN1L/CAMK1D/FAM219B/LINC00852/LTBP4/SOD1/C8orf40/SERTAD4-AS1/LRPAP1/MMGT1/REEP5/RNF220/LOC100286925/CLSTN1/NFE2L1/FAM149A/PLCD3/RADIL/CBX6/SLC29A1/FOXN3/MFGE8/LRP2BP/CGRRF1/ZNF503/IQSEC1/ILF3-AS1/SUN2/FAM43B/ACTN4/VAMP2/CYB5R3/FHL1/FBXW4/EMILIN3/ITPRIPL2/MYOC/HNRNPUL1/ARHGAP12/CCDC3/TMEM41A/SDC4/DPYSL2/MRPL21/PPP1R3C/AFF3/C8orf37/C3orf37/MSTO1/APBB1/FRMD7/SPRYD3/HEY2/DANCR/PEBP1/ARHGAP23/DHX38/TRIM47/PCOLCE2/CTNNA1/DOCK9-AS2/DCTN1/TACC1/CHD9/SORBS3/ZFHX3/CKB/SETD6/FAM107A/COX11/DCUN1D5/FOXF2/AQP1/RERG/NUDC// |  |
| References: 1. Lewis, M.J.; Barnes, M.R.; Blighe, K.; Goldmann, K.; Rana, S.; Hackney, J.A.; Ramamoorthi, N.; John, C.R.; Watson, D.S.; Kummerfeld, S.K.; et al. Molecular Portraits of Early Rheumatoid Arthritis Identify Clinical and Treatment Response Phenotypes. Cell Reports 2019, 28, 2455-2470.e5. 2. Zhong, Q.; Gong, F.-Y.; Gong, Z.; Hua, S.-H.; Zeng, K.-Q.; Gao, X.-M. IgG Immunocomplexes Sensitize Human Monocytes for Inflammatory Hyperactivity via Transcriptomic and Epigenetic Reprogramming in Rheumatoid Arthritis. J.I. 2018, 200, 3913–3925. 4. Martinez, F.O.; Gordon, S.; Locati, M.; Mantovani, A. Transcriptional profiling of the human monocyte-to-macrophage differentiation and polarization: new molecules and patterns of gene expression. J. Immunol. 2006, 177, 7303–7311. 3. Dennis, G.; Holweg, C.T.; Kummerfeld, S.K.; Choy, D.F.; Setiadi, A.; Hackney, J.A.; Haverty, P.M.; Gilbert, H.; Lin, W.; Diehl, L.; et al. Synovial phenotypes in rheumatoid arthritis correlate with response to biologic therapeutics. Arthritis Res Ther 2014, 16, R90. | | | | |
