# Supplementary material for: Disease-Specific Autoantibodies Induce Trained Immunity in RA Synovial Tissues and Its Gene Signature Correlates with the Response to Clinical Therapy
Source: Mediators Inflamm. 2020 Oct 6;2020:2109325. doi: 10.1155/2020/2109325 (PMC7558774; doi:10.1155/2020/2109325)
Supplement: Supplementary Materials — Supplementary Table 1: information of patients. Supplementary Table 2: gene lists used in this study. [file 2109325.f1.zip › supplementary_table1.docx]

**Supplementary Table 1. Information of patients**

| Variables | Healthy donors  (HC) | Osteoarthritis patients  (OA) | Arthralgia patients  (AR) | Undifferentiated arthritis  (UA) | Early rheumatoid arthritis  (eRA) | Established rheumatoid arthritis  (est.RA) |
| --- | --- | --- | --- | --- | --- | --- |
| Numbers of participants | 28 | 22 | 10 | 6 | 57 | 95 |
| Age (years, mean ± SD) | 35.2 ± 16.2 | 49.1 ± 18.9 | 52.5 ± 11.9 | 46 ± 12.8 | 55.9 ± 16.7 | 54 ± 13.6 |
| Female/male | 14/14 | 13/9 | 10/0 | 6/0 | 33/20 | 73/22 |
| ACPA positivity,  n (%) | NA | NA | 10, 100% | 6, 100% | 39, 68.4% | 57, 60% |
| Criteria | Heathy donors were obtained from patients attending a sports medicine day surgical facility with knee pain, while had **NO** evidence of any form of arthritis on history or examination and had NO cartilage damage or synovitis on knee arthroscopy. | Osteoarthritis patients were obtained from patients attending a sports medicine day surgical facility with knee pain, with a clinical history and/or examination findings suggestive of OA in addition to supporting arthroscopic findings. | Arthralgia patients were defined as subjects with symptoms of aches and pains, without clinical signs of synovitis or significantly raised C-reactive protein at first assessment, but with positive RF and ACPA. | Undifferentiated arthritis patients were defined as subjects presenting with clinical signs of synovitis, but who failed to meet the 2010 American College of Rheumatology criteria for RA. | All patients with early RA fulfilled the American College of Rheumatology (ACR) criteria 2010, and were included within 12 months of diagnosis without prior treatment. | All patients with established RA were diagnosed > 1 year before sample collection, and average disease duration was 68 month and all of them had received disease-modifying antirheumatic drugs or anti-TNFα treatments. |
